# Identifying novel transcript biomarkers for hepatocellular carcinoma (HCC) using RNA-Seq datasets and machine learning

**DOI:** 10.1186/s12885-021-08704-9

**Published:** 2021-08-27

**Authors:** Rajinder Gupta, Jos Kleinjans, Florian Caiment

**Affiliations:** Department of Toxicogenomics, School of Oncology and Developmental Biology (GROW), Maastricht University, Maastricht, The Netherlands

**Keywords:** Hepatocellular carcinoma, Machine learning, Biomarkers, RNA-Seq, Transcript expression

## Abstract

**Background:**

Hepatocellular carcinoma (HCC) is one of the leading causes of cancer death in the world owing to limitations in its prognosis. The current prognosis approaches include radiological examination and detection of serum biomarkers, however, both have limited efficiency and are ineffective in early prognosis. Due to such limitations, we propose to use RNA-Seq data for evaluating putative higher accuracy biomarkers at the transcript level that could help in early prognosis.

**Methods:**

To identify such potential transcript biomarkers, RNA-Seq data for healthy liver and various HCC cell models were subjected to five different machine learning algorithms: random forest, K-nearest neighbor, Naïve Bayes, support vector machine, and neural networks. Various metrics, namely sensitivity, specificity, MCC, informedness, and AUC-ROC (except for support vector machine) were evaluated. The algorithms that produced the highest values for all metrics were chosen to extract the top features that were subjected to recursive feature elimination. Through recursive feature elimination, the least number of features were obtained to differentiate between the healthy and HCC cell models.

**Results:**

From the metrics used, it is demonstrated that the efficiency of the known protein biomarkers for HCC is comparatively lower than complete transcriptomics data. Among the different machine learning algorithms, random forest and support vector machine demonstrated the best performance. Using recursive feature elimination on top features of random forest and support vector machine three transcripts were selected that had an accuracy of 0.97 and kappa of 0.93. Of the three transcripts, two were protein coding (PARP2–202 and SPON2–203) and one was a non-coding transcript (CYREN-211). Lastly, we demonstrated that these three selected transcripts outperformed randomly taken three transcripts (15,000 combinations), hence were not chance findings, and could then be an interesting candidate for new HCC biomarker development.

**Conclusion:**

Using RNA-Seq data combined with machine learning approaches can aid in finding novel transcript biomarkers. The three biomarkers identified: PARP2–202, SPON2–203, and CYREN-211, presented the highest accuracy among all other transcripts in differentiating the healthy and HCC cell models. The machine learning pipeline developed in this study can be used for any RNA-Seq dataset to find novel transcript biomarkers.

Code: www.github.com/rajinder4489/ML_biomarkers

**Supplementary Information:**

The online version contains supplementary material available at 10.1186/s12885-021-08704-9.

## Introduction

The liver, one of the largest organ in the body, performs various important functions, such as filtering harmful substances from the blood to be then excreted from the body, producing bile to help in the digestion of fats from food, or storing glycogen (sugar) that will be used for energy. Due to its continuous exposure to harmful substances, it is prone to the amplitude of diseases that can eventually cause liver failure and/or liver cancer. Cirrhosis, long-term infection with hepatitis B virus, and hepatitis C virus, alcoholic liver disease, and nonalcoholic fatty liver disease (NAFLD) are leading risk factors for primary liver cancer [1]. Moreover, cancer can develop in the liver at any stage in the progression of various liver diseases. As published in independent reports by World Health Organization (WHO) [2] and the US Center for Disease Control and Prevention (CDC) [3], liver cancer is among the top causes for cancer death worldwide, of which hepatocellular carcinoma (HCC) is the most common type of primary liver cancer, accounting for ~ 80% liver cancers.

Reducing the global burden of HCC is, therefore, a primary concern and it can be achieved by improving early detection and management [4]. Currently, the employed prognosis for HCC includes radiological examinations and assessment of serum markers. Radiological examinations are limited for early diagnosis as the performance of the imaging techniques begins to degrade substantially below a lesion size of 2 cm and have only modest accuracy below a lesion size of 1 cm [5]. In the case of biomarkers, currently, there are ~ 20 biomarkers (Table 1) in research, and out of these only α-fetoprotein (alpha-fetoprotein or AFP) has a clinical application; even though it is ineffective for detecting early lesions [1, 24–26]. Of the other markers used in research, none have reached the standard level of clinical practice so far [24, 27]. However, in various studies, it has also been demonstrated that a combination of different biomarkers provides higher accuracy in predicting HCC [6, 11, 20–23].

**Table 1:** Currently used serum biomarkers in the prognosis of hepatocellular carcinoma (HCC)

Used as	Biomarker(s)	Name	Comments
Individual biomarkers	AFP [6]	Alpha-fetoprotein	Increased, a sign of liver cancer
	DCP [6]	des-gamma-carboxy prothrombin	Increased, a sign of liver cancer
	GPC3 [7]	Glypican-3	GPC3 is overexpressed in HCC
	GP73 [8]	Golgi glycoprotein 73	High expression of GP73 in primary HCC
	MDK [9]	Midkine	Overexpressed in tumors
	OPN [10]	Osteopontin	Overexpressed
	SCCA [11]	Squamous cell carcinoma antigen	SCCA1, SCCA2 overexpressed
	ANXA2 [12]	Annexin A2	Increased in HCC
	Annexin A7 [13]	Annexin A7	Increased expression inhibits HCC lymph node metastasis
	CD44 [14]	Cluster Differentiation 44	Increased
	CD90 [14]	Cluster Differentiation 90	Increased
	CD133 [15]	Cluster Differentiation 133 or prominin-1	CD133 protein expression levels of HCC in both the cytoplasm and nucleus were significantly higher than adjacent normal liver tissue.
	EpCAM [16]	Epithelial cell adhesion molecule	Tumor size, intrahepatic metastasis, and EpCAM positivity were associated with tumor recurrence
	TGF-β (1,2,3) [17]	Transforming growth factor beta	Highly activated
	FGF [18]	Fibroblast growth factor	Expression was only detected in the liver tissues of patients with chronic hepatitis type C and HCC
	HGF/SF [19]	Hepatocyte growth factor receptor	HGFA and Matriptase convert pro-HGF/SF to mature HGF/SF
Combination of biomarkers	AFP, AFP-L3, DCP [6]	Alpha-fetoprotein, *L. culinaris* agglutinin-reactive fraction of alpha-fetoprotein, des-gamma-carboxy prothrombin	Increased, a sign of liver cancer
	CK19, GPC3, AFP [20]	Cytokeratin 19, Glypican-3, Alpha-fetoprotein	GPC3 with CK19 and AFP
	GPC3, HSP70, GS [21]	Glypican 3, Heat shock protein 70, Glutamine synthetase	All increased, show a better diagnosis
	TLN1, MDK [22]	Talin-1, Midkine	Talin-1 decreased, MDK increased in serum
	SCCA-AFP [11]	Squamous cell carcinoma antigen, Alpha-fetoprotein	Overexpressed
	HIF-1α, VEGF (A-D) [23]	Hypoxia-inducible factor-1α, vascular endothelial growth factor	HIF-1α and VEGF showed higher expression

Though the combinations of various biomarkers are better predictors than the individual biomarkers, sensitivity or specificity is still low for all biomarker combinations [6, 11, 20–23]. While proteins are the major functional element, the corresponding transcripts can be an easier surrogate to detect and quantify. The cancer-specific mRNAs can leak into the serum as a result of passive processes (such as necrosis) and active processes (such as tumor cell apoptosis and active release in microvesicles by tumor cells) [28–31]. Though non-invasive, the lack of transcriptomics data for circulating cell-free mRNAs for HCC poses a limitation in undertaking a comprehensive in silico study to find novel biomarkers in serum. Only one study was found where the extracellular mRNAs for three HCC cell models, namely HepG2, Huh7, and immortalized normal liver PH5CH cells were profiled [32]. On the other hand, exhaustive transcriptomics data is available for HCC tissue/cell models (c.f. Methods) and hence, we concentrated on such data to find novel HCC biomarkers.

Using RNA-Sequencing (RNA-Seq), the whole transcriptome can be quantified. Moreover, different types of transcripts (protein coding and non-coding) can also be identified. Most transcriptomics analyses focus on gene expression by aggregating the expression of all transcripts for the given gene. However, in this study, we will focus on the transcripts because alternative-splicing defects in cancer are well documented [33–35] and dysregulation of splicing variants’ expression has recently emerged as a novel cancer hallmark [35]. Moreover, using the RNA-Seq data at the transcript level will also allow us to investigate the potency of non-coding transcripts to be used as biomarkers.

Machine learning (ML) is a multidisciplinary field that makes use of computer science, artificial intelligence, computational statistics, and information theory to build algorithms that learn from existing data and make predictions on new data [36]. It has found application in diverse domains of biomedicine, including, but not limited to, image analysis [37], cancer prediction from heterogeneous data [38], robust phenotyping [39], gene discovery [40], differential network analysis [41], biomarker discovery [42], and transcriptional regulated genes [43]. The application of machine learning for the biomarker discovery from the RNA-Seq data is mainly focused on genes, however, recent studies have demonstrated that transcript-based analyses outperformed gene-based analyses using ML [44, 45]. To assess if transcript biomarkers have better prediction accuracy, we analyzed various HCC cell models and healthy liver RNA-Seq data. Several HCC cell models were taken for this study (Table 2) to ascertain that their biological heterogeneity is accounted for while building the ML models. Various ML algorithms, namely random forest (RF), K-nearest neighbors (KNN), support vector machines (SVM), Naïve Bayes (NB), and Neural networks (NNET), which are extensively used in the field of biomedicine, were applied to build the models and identify novel putative transcript biomarkers for HCC.

**Table 2:** HCC cell models and healthy liver samples were taken for this study from various studies

ENA			Instrument	Cell model	Type	Number of replicates
Study Id		Run accession				
PRJDB2882		DRR018792	HiSeq 2500	Huh7.5.1	HCC	1
PRJEB27210		ERR2619174, ERR2619175, ERR2619176, ERR2619177	HiSeq 2500	Hep3B	HCC	4
		ERR2619178, ERR2619179, ERR2619180, ERR2619181		HepG2	HCC	4
		ERR2619182, ERR2619183, ERR2619184, ERR2619185		HuH-7	HCC	4
PRJEB27210		ERR2619186, ERR2619187, ERR2619188, ERR2619189, ERR2619190, ERR2619191	HiSeq 2500	PHH	Healthy liver	6
PRJNA357266		SRR5104155	HiSeq 2500	LM3	HCC	1
PRJNA386625		SRR5576264, SRR5576288	HiSeq 2500	HepaRG	HCC	2
PRJNA523380		SRR8615310	HiSeq 2500	SNU-398	HCC	1
		SRR8615311		SNU-387	HCC	1
		SRR8615387		Li-7	HCC	1
		SRR8615471		SNU-878	HCC	1
		SRR8615472		SNU-886	HCC	1
		SRR8615483		JHH-1	HCC	1
		SRR8615650		SNU-475	HCC	1
		SRR8615654		SNU-423	HCC	1
		SRR8615655		SNU-449	HCC	1
		SRR8615661		HuH-7	HCC	1
		SRR8615664		HuH-1	HCC	1
		SRR8615682		SK-HEP-1	HCC	1
		SRR8615914		JHH-7	HCC	1
		SRR8615918		JHH-2	HCC	1
		SRR8615919		JHH-4	HCC	1
		SRR8615920		JHH-5	HCC	1
		SRR8615921		JHH-6	HCC	1
		SRR8615932		SNU-182	HCC	1
		SRR8615968		PLC/PRF/5	HCC	1
		SRR8616023		SNU-761	HCC	1
		SRR8616130		Hep 3B2.1–7	HCC	1
		SRR8616135		HLF	HCC	1
PRJNA206422		SRR873426	HiSeq 2000	HKCI-1	HCC	1
		SRR873427		HKCI-4	HCC	1
		SRR873428		HKCI-7	HCC	1
		SRR873429		HKCI-9	HCC	1
		SRR873430		HKCI-11	HCC	1
		SRR873836		HKCI-5B	HCC	1
EU-ToxRisk	PRJEB35350	ERR3668587, ERR3668588, ERR3668589, ERR3668591, ERR3668592, ERR3668593, ERR3668594, ERR3668595, ERR3668596, ERR3668597, ERR3668598, ERR3668600, ERR3668601, ERR3668602, ERR3668603, ERR3668604, ERR3668605, ERR3668606, ERR3668607, ERR3668609, ERR3668610, ERR3668611, ERR3668612, ERR3668613	NovaSeq 6000	Healthy in vivo liver	Healthy liver	24^a^
	PRJEB24482	ERR2259771, ERR2259772, ERR2259773, ERR2259774, ERR2259775, ERR2259776, ERR2259777, ERR2259778, ERR2259779	HiSeq 2500	Liver microtissues 3D	Healthy liver	9
	PRJEB23590	ERR2203448, ERR2203449, ERR2203450, ERR2203451, ERR2203452, ERR2203453, ERR2203455, ERR2203456, ERR2203457, ERR2203458, ERR2203459	HiSeq 2500	Primary human hepatocytes (PHH)	Healthy liver	11^b^
	PRJEB24484	ERR2259780, ERR2259781, ERR2259782, ERR2259783	HiSeq 2500	Human precision-cut liver slices from HCC patients (hPCLiS)	HCC	4
	PRJEB24487	ERR2260002, ERR2260003, ERR2260004, ERR2260005	HiSeq 2500	HepaRG 3D	HCC	4
	PRJEB24466	ERR2259111, ERR2259112, ERR2259113	HiSeq 2500	HepG2	HCC	7
	PRJEB24464	ERR2259092, ERR2259093, ERR2259094, ERR2259095				

^a^There were a total of 27 samples but three samples from children or infants were removed

^b^There were a total of 12 replicates for PHH, one was removed for low library depth during filtration for quality

From the transcriptomics data, three datasets were assembled: all transcripts, protein coding only, and non-coding only. The goal of making these three datasets was to see if one of them provides a better prediction. Consecutively, the efficiency of the known protein biomarkers (Table 1) was also assessed by taking the transcripts for their corresponding genes. The mapped genes also comprised protein coding and non-coding transcripts and they were also made into three datasets (as given above). The results from the complete transcriptomics data and known protein biomarkers (for all datasets) were compared to establish which dataset(s) performs better.

## Methodology

**Fig. 1:**
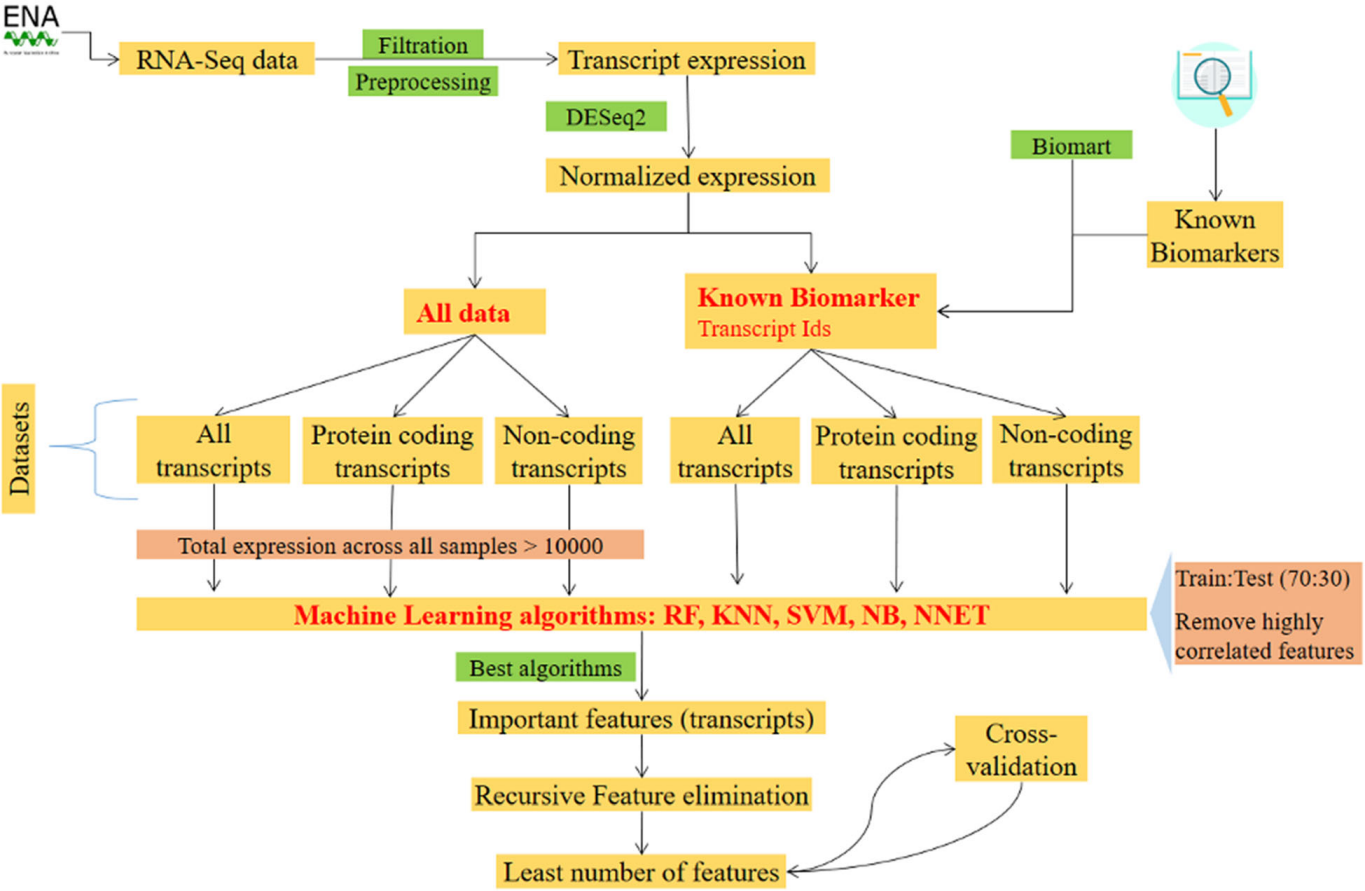
Overview of the workflow: Steps followed to find the least number of features (transcripts) required to identify the transcriptomics biomarkers. Various machine learning algorithms were used, namely random forest (RF), K-nearest neighbor (KNN), Naïve Bayes (NB), support vector machine (SVM), and neural networks (NNET)

The overview of the methodology is presented in Fig. 1 and detailed steps are given below.

1. Data collection

- HCC cell models: The list of all HCC human cell models was obtained from Cellosaurus [46] (Suppl. Table 1).
- RNA-Seq data: Using the names and synonyms of these cell models, RNA-Seq datasets were searched on the European Nucleotide Archive (ENA) and were filtered for baseline expression, instrument model (Illumina HiSeq 2000 or HiSeq 2500 or NovaSeq 6000), and paired-end library layout (Table 2). The samples were also taken from the Horizon 2020 EU-ToxRisk project, as listed in Table 2.

c. Known biomarkers: Concurrently, a list of all known biomarkers for HCC was collected through an exhaustive literature review (Table 1). These biomarkers were mapped to their corresponding Ensembl gene ids using Biomart and manual curation. In instances where there was more than one gene mapping to the protein biomarker, all instances were taken. For all the Ensembl genes that were mapped to the biomarkers, all of them had multiple isoforms/transcripts, comprising of both protein coding and non-coding transcripts.

2. Data preprocessing: The raw RNA-Seq data (fastq files) were first trimmed of their adapter sequences using Trimmomatic [47], mapped onto the human genome (version 84) from Ensembl [48] using Bowtie2 [49], and quantified using RSEM [50]. Isoform read counts were then normalized for different studies using DESeq2 [51].
3. Machine learning:
  - Preparing different datasets: We analyzed the known protein biomarkers and complete data (named as all data) separately. Furthermore, the transcriptomics data consists of protein coding and non-coding transcripts and it provided the opportunity to investigate the efficiency of different types of transcripts in identifying healthy and HCC cell models. We made three datasets, namely all transcripts (protein coding and non-coding), protein coding only, and non-coding only for both – all data and known protein biomarkers (Fig. 1).
  - Machine learning algorithms: On these six datasets (Fig. 1), machine learning algorithms from the caret package in R [52] were applied. We used five different algorithms, namely random forest (RF), K-nearest neighbors (KNN), support vector machines (SVM), Naïve Bayes (NB), and Neural networks (NNET) with ten-fold cross-validation for ten times. All further steps are applied to all six datasets individually. The seed was fixed to have reproducible results.
    The data was first divided into 70:30 for training and testing, respectively. A separate validation set was not created because we used k-fold cross-validation to tune the model’s hyper-parameters. In the case of datasets (all transcripts, protein coding only, and non-coding only) from all data, all transcripts that had a total expression for all samples below 10,000 were removed. This expression filter was applied to take into account only the highly expressed transcripts. However, in the case of known biomarkers, no such filter was used since we wanted to retain all information. Furthermore, using the ‘findCorrelation’ feature from the Caret library, highly correlated transcripts (> 0.75) were identified and removed, except one (the first, a random transcript). Each algorithm’s performance is assessed on all datasets by evaluating various metrics, namely sensitivity, specificity, accuracy, Matthew’s correlation coefficient (MCC), and informedness (eqs. 1–4) using R library ‘MLeval’ [53] (Table 3). All metrics were calculated using the transcript expression. Additionally, the time taken by each algorithm to run is also provided.
    Based on the results from these metrics, the best algorithm and dataset were selected and the top 20 important features (transcripts) were extracted using “varImp” from the Caret library. Then to find the minimum set of features to differentiate between healthy and HCC cell models, “RFE” (Recursive Feature Elimination) from the Caret library was applied using the method cross-validation (CV).
  1 $$Sensitivity\ or\ TPR=\frac{TP}{TP+ FN}$$
  2 $$Specificity\ or\ TNR=\frac{TN}{TN+ FP}$$
  3 $$MCC=\frac{TP. TN- FP. FN}{\sqrt{\left( TP+ FP\right)\left( TP+ FN\right)\left( TN+ FP\right)\left( TN+ FN\right)}}$$
  4 $$Informedness= Sensitivity+ Specificity-1$$
  where.
  TP is true positive.
  TN is true negative.
  FP is false positive.
  FN is false negative.
  MCC is Mathew’s correlation coefficient
4. Re-training the model: The features (transcripts) selected using RFE were used to train the final model. Taking these features, exhaustive k-fold cross-validation was run by setting the repeats to 100 and number to 10; implying 1000 instances will be evaluated.
5. Chance findings: There were a total of ~ 200 k transcripts and to establish that the features (transcripts) selected using RFE were not chance findings, 15,000 iterations were performed taking three random transcripts out of the highly expressed transcripts to compare their prediction accuracy. The results from randomly taken transcripts were compared to the selected features (transcripts from RFE).

**Table 3:** Number of transcripts after steps of filtration and time to run ML algorithms on them

		Datasets					
		Known protein biomarkers			All data		
Steps		All transcripts	Protein coding	Non-coding	All transcripts	Protein coding	Non-coding
Number of transcripts after expression filter; biomarkers no filter, all data > 10,000		410	262	149	16,173	13,688	2724
Number of highly correlated features (transcripts); correlation cutoff > 0.75		177	98	37	12,047	9866	1970
Number of transcripts after removing highly correlated features		234	165	113	4127	3823	755
Time to run (*in seconds*)	RF	10.77	8.09	6.44	196.25	169.31	32.60
	NB	12.34	9.38	6.63	297.81	280.27	46.05
	KNN	1.03	1.10	1.11	5.63	5.62	1.78
	SVM	2.25	1,07	1.05	7.51	7.48	2.72
	NNET	72.37	35.84	20.12	71,044.53	56,114.75	3125.74

## Results

To obtain an exhaustive list of all HCC in vitro cell models, Cellosaurus [46] was used (accessed on 27/08/2019). It houses data for 250 HCC cell models for humans (Suppl. Table 1). RNA-Seq data for all 250 cell models were searched on ENA using the application programming interface (API), taking the data generated using Illumina’s HiSeq platforms or newer and library layout as paired-end. Furthermore, it was manually checked if the data were obtained at baseline. A total of 51 samples from 6 studies comprising of 33 cell models from ENA passed the filters and manual curation (Table 1). Samples from the EU-ToxRisk project were also taken; healthy in vivo liver (24 samples) and all other samples (32 samples from 5 cell models) were sequenced on NovaSeq 6000 and HiSeq 2500, respectively (Table 1).

The samples’ quality was assessed using FastQC, and it was observed that all samples passed the “Per base sequence quality” metric. However, one sample (PHH_024_1) did not pass the library size filter and was discarded. The samples passing the filters were then processed and the transcript expression was normalized using DESeq2 for different studies.

We first investigated the expression patterns of the known biomarkers at the transcript level to see if the protein coding transcripts demonstrate a similar expression pattern as known protein biomarkers. Each gene can have multiple protein coding transcripts, only the ones mapped to manually annotated and reviewed Uniprot identifiers were considered and their expression pattern was examined (Suppl. Fig. 1). VEGFA-223, HSP90AB1–203, FGF5–201, ANXA7–201, and SPP1–201 were the most down-regulated and CD44–206, HSP90AB1–201, SPP1–202, ANXA2–202, and CD44–209 were the most upregulated transcripts.

We then investigated the accuracy of the known biomarkers (all three datasets, namely all transcripts, protein coding only, non-coding only) and all data (all three datasets), in predicting the correct labels for the cell models. We focused only on highly expressed transcripts and hence, to remove the lowly expressed ones, an expression filter was introduced (total expression across all samples > 10,000 reads) (Table 3). However, in the case of known biomarkers, no such filter was used because we wanted to preserve any information, if present, held by even the lowly expressed transcripts. Furthermore, all transcripts having a high correlation (> 0.75) were discarded to remove redundancy except the first (random) transcript in the list. To the remaining transcripts in each dataset, ML algorithms were applied, individually. While KNN and SVM were the fastest to run (a few seconds), NNET took the longest time for all datasets (most for all data-all transcripts: ~ 19 h 44 min) (Table 3).

**Fig. 2:**
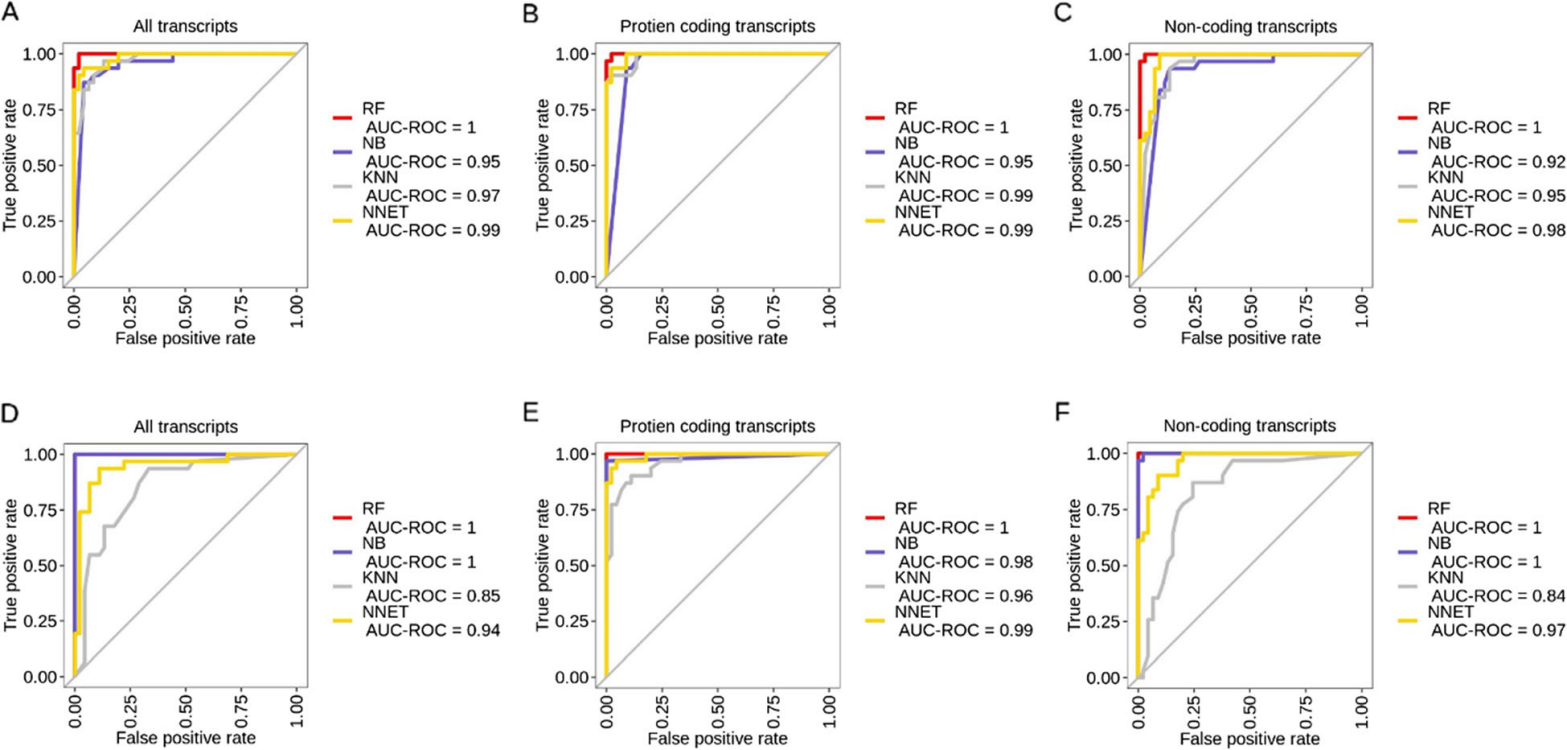
AUC-ROC: The area under the ROC (receiver operating characteristic) curve for all datasets analyzed using different machine learning algorithms, namely K-nearest neighbors (KNN), Naïve Bayes (NB), neural network (NNET), and random forest (RF). Known biomarker (**A**: all transcripts, **B**: protein coding transcripts, and C: non-coding transcripts) and all data (**D**: all transcripts, E: protein coding transcripts, and F: non-coding transcripts). For all the calculations, transcript expression was used

**Fig. 3:**
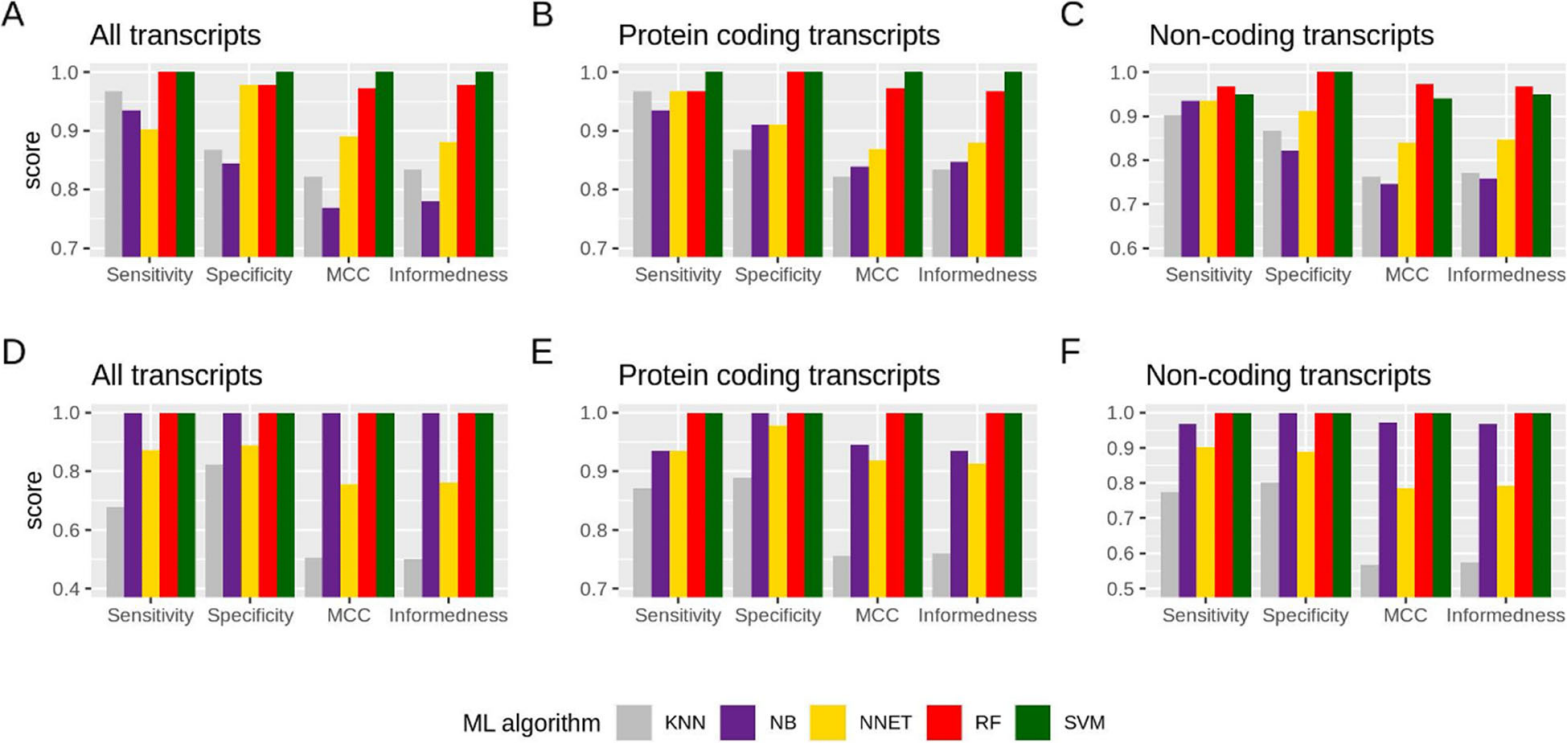
Machine learning (ML) metrics values. The values for different metrics calculated for all datasets using different machine learning algorithms, namely K-nearest neighbors (KNN), Naïve Bayes (NB), neural network (NNET), random forest (RF), and support vector machine (SVM). Known biomarker (**A**: all transcripts, **B**: protein coding transcripts, and **C**: non-coding transcripts) and all data (**D**: all transcripts, **E**: protein coding transcripts, and F: non-coding transcripts). For all the calculations, transcript expression was used

**Fig. 4:**
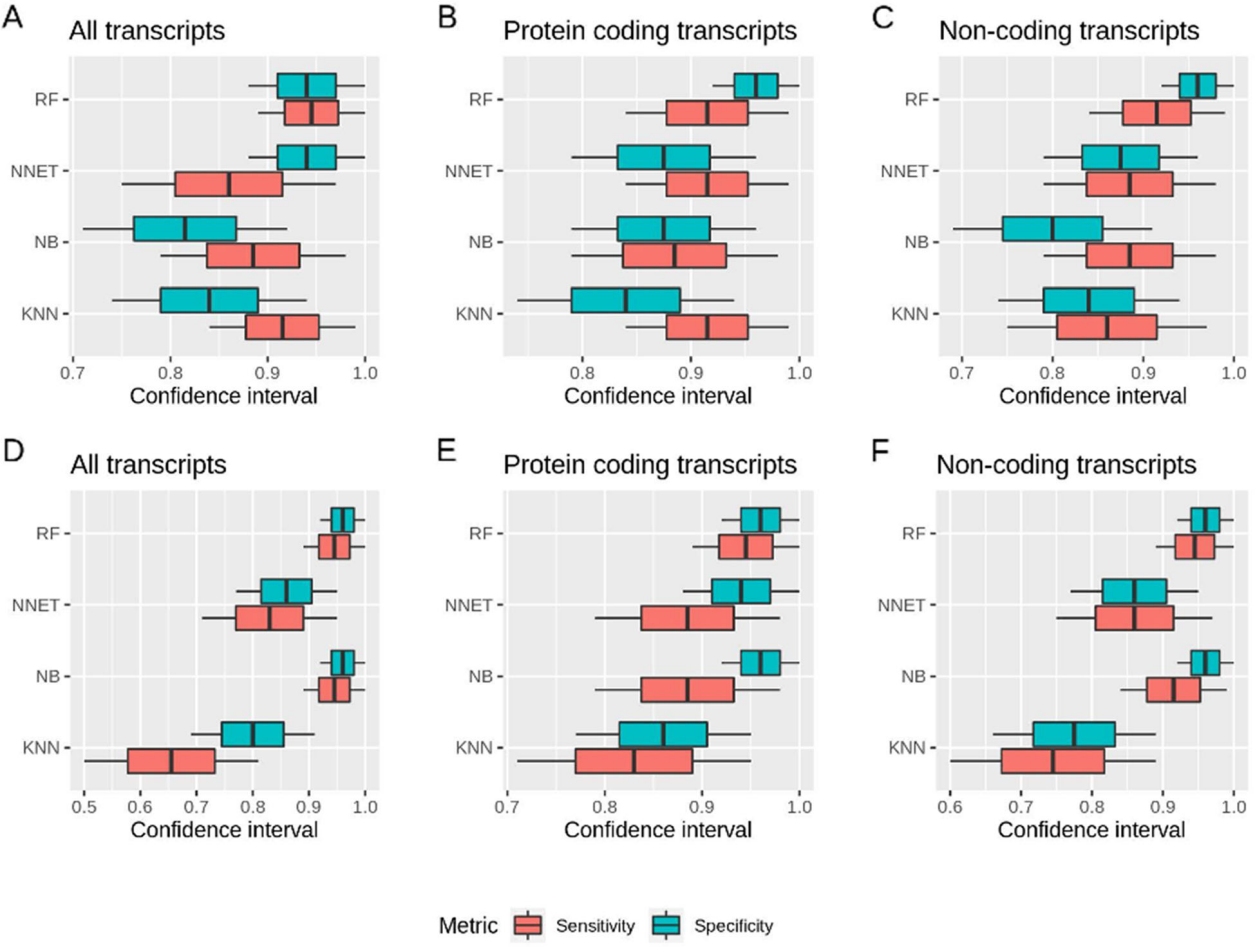
Confidence interval: Confidence interval for sensitivity and specificity across all machine learning algorithms, namely K-nearest neighbors (KNN), Naïve Bayes (NB), neural network (NNET), and random forest (RF). Known biomarker (**A**: all transcripts, **B**: protein coding transcripts, and **C**: non-coding transcripts) and all data (**D**: all transcripts, E: protein coding transcripts, and F: non-coding transcripts). For all the calculations, transcript expression was used

The results obtained from the algorithms show that the area under the curve-receiver operating characteristics (AUC-ROC) values was the highest for RF and the lowest for KNN, across all datasets (Fig. 2). AUC-ROC values for SVM cannot be obtained because it is a discrete classifier. For other metrics (sensitivity, specificity, informedness, and MCC) for all datasets, SVM illustrated the highest values (Fig. 3). In the case of known biomarkers, RF demonstrated high values comparable to SVM in some cases for all datasets. NB also illustrated high values for all metrics for all data-all transcripts. We were also interested to see if protein coding or non-coding individually could give a better prediction. However, it was noted that predictions were less accurate when using them separately, as compared to all transcripts. The confidence intervals for sensitivity and specificity were the smallest in the case of all data-all transcripts for all algorithms and particularly for RF and NB (Fig. 4).

**Fig. 5:**
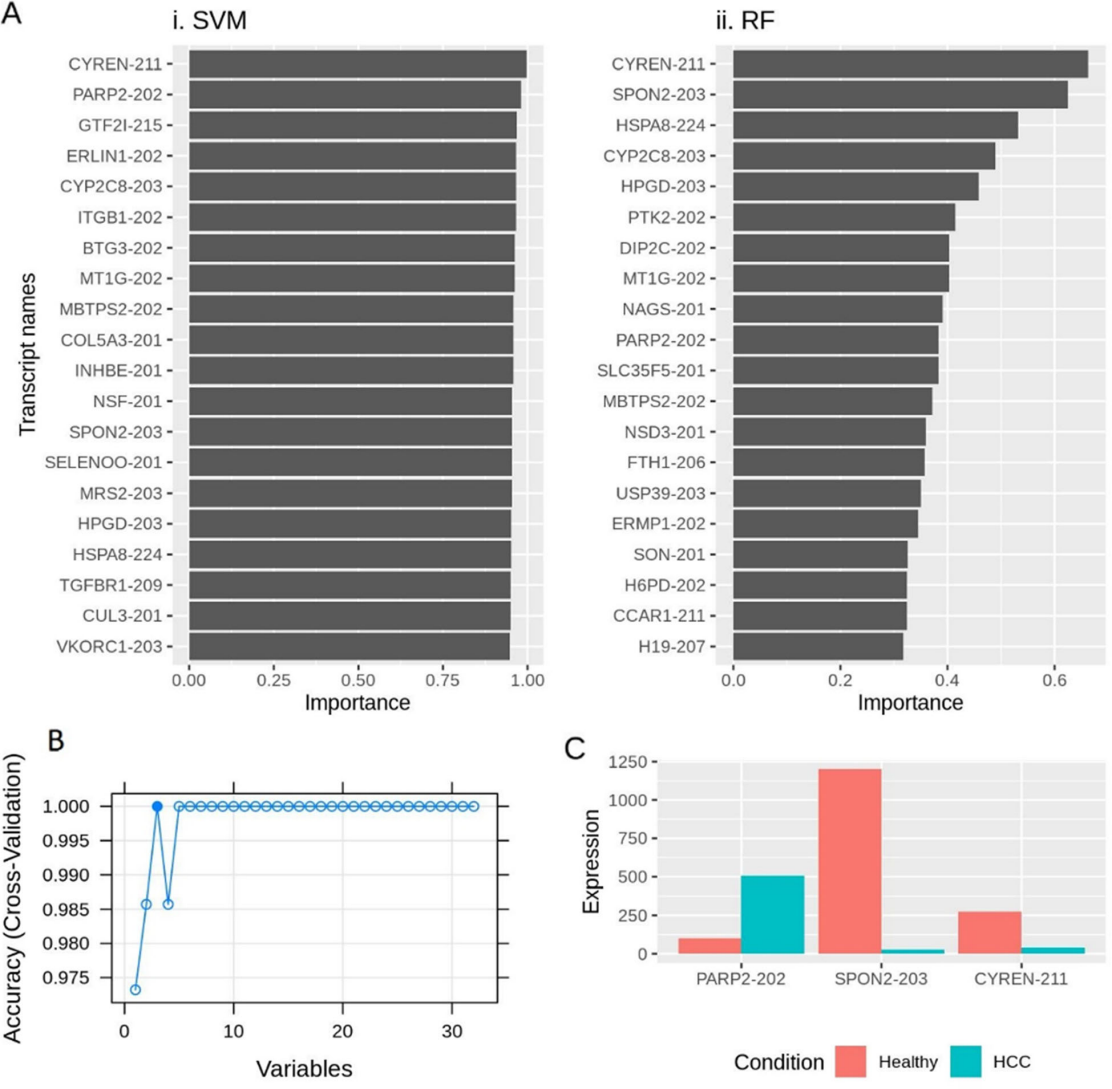
**A** Top 20 important features extracted from all data-all transcripts dataset obtained using (i) SVM and (ii) RF. **B** Recursive feature elimination (RFE) was used with the top 20 features from (**A**) to extract a list of the least number of features required to differentiate between healthy and HCC cell models. Three features were selected having an accuracy of 0.97 and kappa of 0.93 (**C**) Average expression of three features (transcripts), across healthy liver and HCC cell models, chosen in (**B**)

Based on the values of different metrics used to assess the performance of the algorithms on various datasets, RF and SVM performed the best for all datasets; primarily for all transcripts, protein coding transcripts, and non-coding transcripts datasets for all data. To further get the least number of features required to differentiate between the healthy and HCC cell models, the top 20 important features (transcripts) from RF and SVM when applied to all data-all transcripts were taken (Fig. 5A). There was a total of 32 unique features (transcripts), with an overlap of eight features between the two algorithms (Suppl. Fig. 2). Furthermore, recursive feature elimination (RFE) was applied to this list to extract the least number of features required to differentiate between healthy and HCC samples. With the application RFE, three features (transcripts) were identified (Fig. 5B), namely PARP2–202 (protein coding transcript), SPON2–203 (protein coding transcript), and CYREN-211 (non-coding transcript) with an accuracy of 0.97 and kappa of 0.93. These three transcripts were present in both algorithm’s top important features. While PARP2–202 was upregulated (log2 fold change: 2.368), SPON2–203 and CYREN-211 were both down-regulated (− 5.421 and − 2.771, respectively) (Fig. 5C).

The PARP2–202 transcript is the second-largest protein coding transcript from the PARP2 gene. It shares 97.8% coding sequence (CDS) identity and similarity with the longest protein coding transcript (PARP2–201) from the same gene. In the case of SPON2–203, it is the largest protein coding transcript from the SPON2 gene [54]. Lastly, CYREN-211 is an 844 bp long non-coding transcript. As PARP2–202 is highly similar and identical to the longest protein coding transcript of the PARP2 gene, it can be assumed that the annotations from the PARP2 gene can be used for PARP2–202. For SPON2–203, it being the longest protein coding transcript for the gene it is the primary gene product. However, for CYREN-211, no annotations could be derived as it is a non-coding transcript and no functional properties are yet defined for it.

An investigation of the gene ontology terms (biological process) obtained using DAVID [55] highlighted that CYREN is involved in double-strand break repair via non-homologous end-joining (GO:0006303) and PARP2 had a known function in DNA repair (GO:0006281), base-excision repair (GO:0006284) and DNA ligation involved in DNA repair (GO:0051103). In the case of SPON2, multiple ontologies for immune responses were obtained – GO:0002448 (mast cell mediated immunity), GO:0008228 (opsonization), GO:0032755 (positive regulation of interleukin-6 production), GO:0032760 (positive regulation of tumor necrosis factor production), GO:0043152 (induction of bacterial agglutination), GO:0045087 (innate immune response), GO:0050832 (defense response to fungus), GO:0051607 (defense response to virus), GO:0060907 (positive regulation of macrophage cytokine production), GO:0071222 (cellular response to lipopolysaccharide), GO:0001530 (lipopolysaccharide binding), and GO:0003823 (antigen binding). The aberrant activation of the DNA repair pathways is linked to various cancers [56, 57] and in recent studies, immune dysfunction in HCC and immunomodulation have been highlighted as a major factor in HCC development [58, 59].

To assess the strength of the model, it was re-trained using the three predicted features (transcripts) but with an increased number of cross-validations (repeats = 100, number = 10; implying 1000 iterations). High values for all metrics were observed with RF and SVM (sensitivity: 0.968 and 0.944 (RF and SVM), specificity: 1 and 1, MCC: 0.973 and 0.936, informedness: 0.968 and 0.944, and AUC-ROC: 0.99 (RF only)). Moreover, the confidence interval for sensitivity and specificity in the case of RF was 0.84–0.99 and 0.92–1, respectively. Finally, to establish that these transcripts (PARP2–202, SPON2–203, and CYREN-211) were not chance findings, random combinations of three transcripts (highly expressed) were made and their efficiency was assessed and compared to the three transcripts selected using RFE. It was observed that out of 15,000 combinations created, none of the combinations exhibited higher or equal values for the metrics for RF and only 0.12% cases (18 cases) demonstrated higher or equal value for the metrics in the case of SVM (Suppl. Table 2).

## Discussion

Hepatocellular carcinoma (HCC) has a huge global burden and the challenge lies primarily in its early detection owing to the limited accuracy of serum biomarkers and inefficiency of radiological examinations. With advancements made in machine learning over the last few years, we investigated if it can assist in finding better biomarkers for HCC. We took RNA-Seq data from HCC and healthy liver cell models and used various machine learning algorithms to highlight key features that can differentiate between the healthy and HCC cell models with high accuracy. A set of three transcripts were identified, namely PARP2–202, SPON2–203, and CYREN-211; proposed as novel putative transcript biomarkers.

Though widely studied, RNA-Seq data for HCC at baseline is not abundantly available. Out of 250 HCC cell models listed in Cellosaurus, data could only be obtained for 33 cell models. Many studies were discarded in the process of selection due to single-end library layout, low coverage, exposure to drugs various treatments, and insufficient metadata. For the 33 cell models taken in this study, 28 had only one replicate. This could have been a limiting factor if these were to be analyzed per cell model, however, in this study the focus was on HCC and all cell models were combined to define the transcriptome profile of HCC. Using the transcriptome profile, the cell type and/or condition (healthy/disease/treatment) can then be accurately assessed [60] and then comparing these profiles, distinct features for these profiles can be established.

For HCC, many biomarkers are extensively studied (Table 1), AFP being one of the most studied biomarkers. Although these biomarkers have been established through studies of serum, most of them are predominantly secreted by the liver [61]. In an attempt to compare the efficiency of these known biomarkers and all data with respect to their ability to discriminate between the healthy and HCC cell models, we observed that all data out-performed known biomarkers’ datasets. The comparatively lower accuracy obtained using known biomarkers can be attributed to fewer features (transcripts) in the dataset. While all data constituted of ~ 200 k transcripts, known biomarkers amounted for ~ 400 features only. The transcriptomics data also provided an opportunity to investigate if protein coding or non-coding transcripts could individually be enough to classify healthy and HCC cell models. A loss of information can be witnessed in both instances compared to both types of transcripts taken together (all transcripts datasets) in the case of known biomarkers and all data. This exhibits that the non-coding transcripts are equally important as the protein coding transcripts. Moreover, in recent studies, the dysregulation of long non-coding RNA in HCC has been studied [62] and their use as biomarkers has also been investigated [63].

Multiple machine learning algorithms (RF, NB, SVM, KNN, and NNET) were used to analyze the data, and all exhibited high efficiency. It was surprising to see how well these algorithms performed, despite significant variations in the sample and library preparation by different labs. Though all exhibited high efficiency, we observed some differences among them across all datasets as illustrated by various metrics calculated for them (Figs. 2 and 3). The reason for the varying performance of these algorithms on the same datasets can be explained by how their hyper-parameters are set. For instance, in the case of RF, the hyperparameters can be the number of samples required to split a node or tree depth; for KNN it can be the number of iterations to form k-groups or clusters; for NNET it can be node weights.

The highest values for all metrics were demonstrated by RF and SVM on all data-all transcripts dataset and the confidence intervals were smallest for RF for the mentioned dataset. NB also exhibited high values for all metrics for all data-all transcripts dataset however it performed poorly for other datasets and hence was not considered for further analyses. Hence top 32 important features were extracted from the algorithm-dataset combination (RF and SVM with all data-all transcripts) to find the least number of features using RFE. RFE employs a backward selection of the predictors, starting with all and removing the ones with the least importance in the model. Three transcripts were identified with maximum accuracy and kappa (Fig. 5B). None of these three transcripts were the ones that were taken randomly from correlated transcripts (c.f. Methodology 3b) and hence no transcript was discarded (correlation > 0.75) that could have provided the same prediction accuracy. One of the chosen transcripts was a non-coding transcript (CYREN-211). While many studies have emphasized the role of non-coding transcripts in the initiation, progression, and metastasis of HCC [64–67], their identification as key features to differentiate HCC and healthy liver is highlighted in only a handful of recent studies [68, 69].

Re-training the model using the three selected transcripts by applying exhaustive cross-validation helped in establishing their potency in discriminating the healthy from the HCC cell models. A final comparison with randomly selected highly expressed transcripts further established that these three transcripts were not chance findings; with values for all metrics always higher than the random combinations for RF and only 6 cases exhibited higher values for SVM.

The catalytic activity of PARP2, one of the poly-ADP-ribose polymerase (PARP) enzymes, has been shown to be induced by DNA-strand breaks. This provides evidence for its cellular response to DNA damage [70]. Furthermore, the expression of the PARP enzymes is upregulated in HCC and other tumors [71]; also shown in our results (Fig. 5C). The higher expression of the PARP2 is significantly correlated with larger tumor size, capsular or vascular invasion, lymph node metastasis, and high histological grade [72]. Moreover, high PARP2 expression is correlated with a low 5-year survival rate, however, given the design of the study (cell-models) survival rates could not be determined. In recent years, immune response and modulation of the innate immune system have also been linked to PARP2 [73]. The role of PARP2 in thymocyte development and B-cell lymphopenia are some of the well-studied processes [74, 75]. A reduction in tumor growth in PARP2-deficient host-mice, compared to wild-type specimens (C57 and Balb/c) has also been associated with the immunomodulatory role of PARP2 [76, 77].

While SPON2 knockdown cell lines exhibit higher hepatoma cell migration and invasion, overexpression repressed them [78]. At the immune system level, it promotes infiltration of M1-like macrophages and inhibits tumor metastasis by activating the SPON2-α5β1 integrin signaling that in turn inactivates RhoA and prevents F-actin assembly [79]. SPON2 levels correlated positively with HCC prognosis; it should be mentioned here that the expression of SPON2–203 (Fig. 5C) is for the transcript and not gene/protein. The role of CYREN-211 in HCC could not be evaluated due to the unavailability of the functional annotation of the non-coding transcripts. However, at the gene/protein level, its role in DNA repair by inhibiting classical non-homologous end-joining and thereby promoting error-free repair by homologous recombination in cell cycle phases where sister chromatids are present are well studied [80].

Though these transcripts are validated through in silico approaches and their role in HCC are defined in the literature, an extensive validation in the HCC patients still needs to be done. If established, such an approach can also be used to identify transcript-level biomarkers for various diseases and conditions, thus providing us an opportunity to look beyond proteins and maybe help in the identification of the disease or the condition at an early stage. One drawback of the current study was that the data was taken from the liver and to predict HCC, an invasive approach has to be taken to extract the sample. To look for transcript biomarkers for HCC that are non-invasive, data from HCC patient’s blood serum/plasma will be required. At this moment, the scarcity of such data limits us from exploring the circulating mRNAs from HCC to find novel and potent biomarkers through in silico approaches. A thorough follow-up study would be required to look for non-invasive/circulating transcript biomarkers in the blood of the HCC patients, by generating and analyzing the data as discussed in this study.

## Conclusion

In our investigation of the healthy liver and various HCC cell models to find novel biomarkers, we analyzed RNA-Seq data using machine learning. Comparing the known HCC biomarkers with all other possible transcripts, we first concluded that using the exhaustive transcript list displayed better accuracy, thus implying that better biomarkers exist. Similarly, between all existing transcripts, protein coding transcripts only, or non-coding transcripts only, it was illustrated that all transcriptomics data improved also the overall accuracy. From this observation, it can be concluded that both protein coding and non-coding transcripts hold important information and are regulated under internal and/or external stimuli. This is further supported by the identification of two protein coding (PARP2–202 and SPON2–203) and one non-coding (CYREN-211) transcript as novel and potent biomarker for HCC. However, the findings would have to be validated in vivo.

The pipeline developed in this study to identify transcript level biomarkers for HCC can be applied to other RNA-Seq datasets as well.

## Supplementary Information

**Additional file 1: Fig. S1**: Log2FC for protein-coding transcripts of the known biomarkers. The known biomarkers were mapped to Ensembl gene ids and for these genes, log2FC for the longest protein-coding transcript (healthy liver versus HCC) was observed. It can be seen that most transcripts demonstrate upregulation as established by protein assays. The genes where two or more transcripts were the longest protein-coding, all of them were taken. **Fig. S2**: Overlap between important features: Top 20 important features were taken from RF and SVM for all data-all transcripts. A total of 8 features overlapped between the two algorithms.

**Additional file 2: Suppl. Table 1**: The list of all HCC human cell models that were obtained from Cellosaurus.

**Additional file 3: Suppl. Table 2**: Random combinations of three transcripts to assess their accuracy for correctly identifying the healthy and HCC cell models using ML algorithms.

## Data Availability

All data generated or analyzed during this study are included in this published article [and its supplementary information files]. The RNA-Seq data used is available on European Nucleotide Archive (ENA; https://www.ebi.ac.uk/ena/browser/home); their project and run accession ids are provided in Table 2.

## Abbreviations

AUC-ROC: Area under the curve-receiver operating characteristics
FN: False negative
FP: False positive
HCC: Hepatocellular carcinoma
hPCLiS: Human precision-cut liver slices from HCC patients
KNN: K-Nearest neighbors
MCC: Mathew’s correlation coefficient
ML: Machine learning
NB: Naïve bayes
NNET: Neural networks
PHH: Primary human hepatocytes
RF: Random forest
RNA-Seq: RNA sequencing
SVM: Support vector machine
TN: True negative
TP: True positive
