# Supplementary material for: Identifying novel transcript biomarkers for hepatocellular carcinoma (HCC) using RNA-Seq datasets and machine learning
Source: BMC Cancer. 2021 Aug 27;21:962. doi: 10.1186/s12885-021-08704-9 (PMC8394105; doi:10.1186/s12885-021-08704-9)
Supplement: Supplementary file 1 — Additional file 1: Fig. S1: Log2FC for protein-coding transcripts of the known biomarkers. The known biomarkers were mapped to Ensembl gene ids and for these genes, log2FC for the longest protein-coding transcript (healthy liver versus HCC) was observed. It can be seen that most transcripts demonstrate upregulation as established by protein assays. The genes where two or more transcripts were the longest protein-coding, all of them were taken. Fig. S2: Overlap between important features: Top 20 important features were taken from RF and SVM for all data-all transcripts. A total of 8 features overlapped between the two algorithms. [file 12885_2021_8704_MOESM1_ESM.docx]

**Identifying novel transcript biomarkers for hepatocellular carcinoma (HCC) using RNA-Seq datasets and machine learning**

Rajinder Gupta^1^, Jos Kleinjans^1^, Florian Caiment^1*^

1. Department of Toxicogenomics, School of Oncology and Developmental Biology (GROW), Maastricht University, Maastricht, The Netherlands

*Corresponding author: florian.caiment@maastrichtuniversity.nl


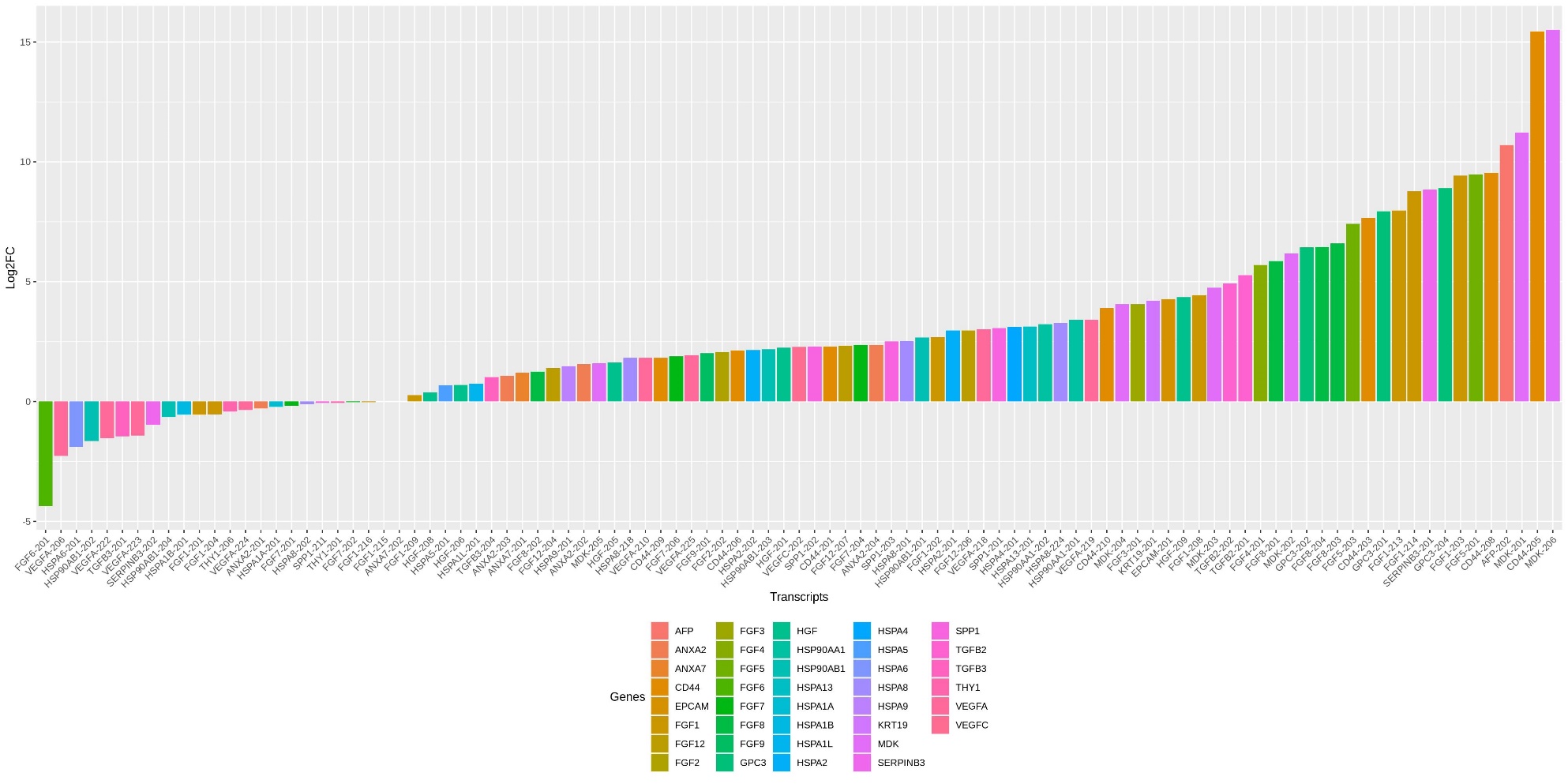


Suppl. Figure 1: **Log2FC for protein-coding transcripts of the known biomarkers**. The known biomarkers were mapped to Ensembl gene ids and for these genes, log2FC for the longest protein-coding transcript (healthy liver versus HCC) was observed. It can be seen that most transcripts demonstrate upregulation as established by protein assays. The genes where two or more transcripts were the longest protein-coding, all of them were taken.


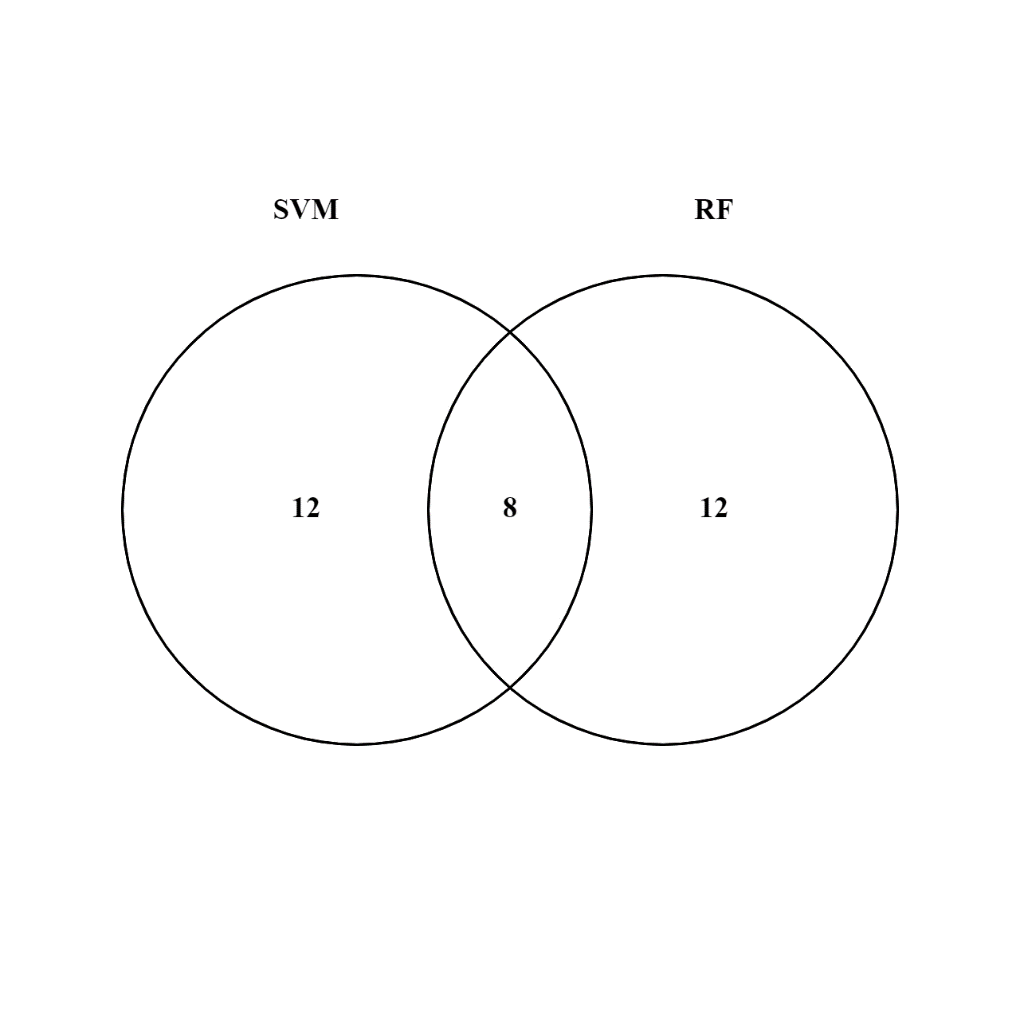


Suppl. Figure 2: **Overlap between important features**: Top 20 important features were taken from RF and SVM for all data-all transcripts. A total of 8 features overlapped between the two algorithms.
